# Supplementary material for: Impaired Height Growth Associated with Vitamin D Deficiency in Young Children from the Japan Environment and Children’s Study
Source: Nutrients. 2022 Aug 13;14(16):3325. doi: 10.3390/nu14163325 (PMC9415323; doi:10.3390/nu14163325)
Supplement: Supplementary file 1 [file nutrients-14-03325-s001.zip › nutrients-1830025_Table_S1.pdf]

Table S1: Profile of the Sub-cohort study

	**Sub-Cohort study** (*n*=3624)		
		(SD)	(95% CI)
Sex			
Male (%)	1828 (50.4)		
Female (%)	1796 (49.6)		
Birth information			
pregnancy period [weeks]	39.53	(1.12)	(39.50, 39.57)
birth weight [g]	3079.3	(378.2)	(3067.0, 3091.7)
birth height [cm]	49.17	(1.96)	(49.11, 49.23)
At 2 years of age			
height [cm]	83.94	(2.93)	(83.85, 84.04)
height SDS	-0.32	(0.98)	(-0.36, -0.29)
weight [kg]	11.52	(2.08)	(11.46, 11.59)
weight SDS	0.12	(0.98)	(0.09, 0.15)
BMI	16.33	(2.70)	(16.24, 16.41)
BMI SDS	0.49	(1.00)	(0.46, 0.52)
At 4 years of age			
height [cm]	99.86	(3.74)	(99.74, 99.98)
height SDS	-0.03	(0.97)	(-0.06, 0.00)
weight [kg]	15.5	(1.77)	(15.45, 15.56)
weight SDS	0.01	(0.88)	(-0.02, 0.04)
BMI	15.52	(1.14)	(15.48, 15.55)
BMI SDS	0.11	(0.84)	(0.08, 0.13)

SD: standard deviation, 95% CI: 95% confidence interval, SDS: standard deviation score, BMI: body mass index
