# Supplementary material for: Impaired Height Growth Associated with Vitamin D Deficiency in Young Children from the Japan Environment and Children’s Study
Source: Nutrients. 2022 Aug 13;14(16):3325. doi: 10.3390/nu14163325 (PMC9415323; doi:10.3390/nu14163325)
Supplement: Supplementary file 1 [file nutrients-14-03325-s001.zip › nutrients-1830025_Table_S2.pdf]

Table S2: Sun exposure habit of each subgroup by serum 25(OH)D3 concentrations

	Subgroup by serum 25(OH)D3 concentrations													
25(OH)D3 [ng/ml]	<10		≥10 to <15		≥15 to <20		≥20 to <25		≥25 to <30		≥30 to <40		≥40	
‥	(*n*=40)		(*n*=254)		(*n*=524)		(*n*=913)		(*n*=925)		(*n*=846)		(*n*=122)	
Time spent playing outside in summer		(%)		(%)		(%)		(%)		(%)		(%)		(%)
rare	0	(0.0)	5	(2.2)	13	(2.6)	14	(1.7)	13	(1.5)	7	(0.9)	1	(0.9)
less than 1 hour	10	(29.4)	39	(16.8)	90	(18.1)	143	(16.9)	182	(20.8)	147	(18.5)	24	(20.9)
1hour or more and less than 3 hours	23	(67.6)	170	(73.3)	359	(72.2)	604	(71.6)	588	(67.0)	561	(70.7)	77	(67.0)
3 hours or more	1	(2.9)	18	(7.8)	35	(7.0)	83	(9.8)	94	(10.7)	79	(9.9)	13	(11.3)
unknown	6		22		27		69		48		52		7	
Time spent playing outside in winter														
rare	8	(23.5)	23	(10.1)	50	(10.2)	61	(7.3)	63	(7.3)	48	(6.2)	3	(2.8)
less than 1 hour	14	(41.2)	104	(45.6)	208	(42.7)	351	(41.9)	353	(40.8)	272	(34.9)	35	(32.4)
1hour or more and less than 3 hours	12	(35.3)	100	(43.9)	212	(43.5)	396	(47.3)	405	(46.8)	424	(54.4)	63	(58.3)
3 hours or more	0	(0.0)	1	(0.4)	17	(3.5)	30	(3.6)	45	(5.2)	35	(4.5)	7	(6.5)
unknown	6		26		37		75		59		67		14	
Frequently wear a hat outside in summer														
yes	29	(85.3)	215	(90.3)	426	(84.0)	740	(85.5)	754	(84.2)	700	(85.9)	95	(81.2)
no	5	(14.7)	23	(9.7)	81	(16.0)	125	(14.5)	141	(15.8)	115	(14.1)	22	(18.8)
unknown	6		16		17		48		30		31		5	
Frequently use a sunscreen in summer														
yes	10	(29.4)	75	(31.5)	142	(28.0)	238	(27.5)	226	(25.3)	253	(31.0)	29	(24.8)
no	24	(70.6)	163	(68.5)	365	(72.0)	628	(72.5)	667	(74.7)	563	(69.0)	88	(75.2)
unknown	6		16		17		47		32		30		5	
